# The effect of different dosing regimens of motesanib on the gallbladder: a randomized phase 1b study in patients with advanced solid tumors

**DOI:** 10.1186/1471-2407-13-242

**Published:** 2013-05-16

**Authors:** Lee S Rosen, Lara Lipton, Timothy J Price, Neil D Belman, Ralph V Boccia, Herbert I Hurwitz, Joe J Stephenson Jr, Lori J Wirth, Sheryl McCoy, Yong-jiang Hei, Cheng-Pang Hsu, Niall C Tebbutt

**Affiliations:** 1 Department of Medicine, University of California Los Angeles, Santa Monica, CA, USA; 2 Western Hospital, Footscray, and Royal Melbourne Hospital, Parkville, VIC, Australia; 3 The Queen Elizabeth Hospital, University of Adelaide School of Medicine, Woodville, SA, Australia; 4 Oncology Hematology of Lehigh Valley, Bethlehem, PA, USA; 5 Center for Cancer and Blood Disorders, Bethesda, MD, USA; 6 Duke University Medical Center, Durham, NC, USA; 7 Cancer Centers of the Carolinas, Greenville, SC, USA; 8 Dana-Farber Cancer Institute and Massachusetts General Hospital, Boston, MA, USA; 9 Department of Biostatistics, Amgen Inc., South San Francisco, CA, USA; 10 Department of Oncology, Amgen Inc., Thousand Oaks, CA, USA; 11 Department of Pharmacokinetics & Drug Metabolism, Amgen Inc, Thousand Oaks, CA, USA; 12 Ludwig Oncology Unit, Austin Hospital, Heidelberg, VIC, Australia

## Abstract

**Background:**

Gallbladder toxicity, including cholecystitis, has been reported with motesanib, an orally administered small-molecule antagonist of VEGFRs 1, 2 and 3; PDGFR; and Kit. We assessed effects of motesanib on gallbladder size and function.

**Methods:**

Patients with advanced metastatic solid tumors ineligible for or progressing on standard-of-care therapies with no history of cholecystitis or biliary disease were randomized 2:1:1 to receive motesanib 125 mg once daily (Arm A); 75 mg twice daily (BID), 14-days-on/7-days-off (Arm B); or 75 mg BID, 5-days-on/2-days-off (Arm C). Primary endpoints were mean change from baseline in gallbladder size (volume by ultrasound; independent review) and function (ejection fraction by CCK-HIDA; investigator assessment).

**Results:**

Forty-nine patients received ≥1 dose of motesanib (Arms A/B/C, n = 25/12/12). Across all patients, gallbladder volume increased by a mean 22.2 cc (from 38.6 cc at baseline) and ejection fraction decreased by a mean 19.2% (from 61.3% at baseline) during treatment. Changes were similar across arms and appeared reversible after treatment discontinuation. Three patients had cholecystitis (grades 1, 2, 3, n = 1 each) that resolved after treatment discontinuation, one patient developed grade 3 acute cholecystitis requiring cholecystectomy, and two patients had other notable grade 1 gallbladder disorders (gallbladder wall thickening, gallbladder dysfunction) (all in Arm A). Two patients developed de novo gallstones during treatment. Twelve patients had right upper quadrant pain (Arms A/B/C, n = 8/1/3). The incidence of biliary “sludge” in Arms A/B/C was 39%/36%/27%.

**Conclusions:**

Motesanib treatment was associated with increased gallbladder volume, decreased ejection fraction, biliary sludge, gallstone formation, and infrequent cholecystitis.

**Trial registration:**

ClinicalTrials.gov NCT00448786

## Background

A key goal of early-phase studies of investigational cancer therapeutics is an assessment of the treatment’s toxicity [1]. However, such studies may be poorly powered to assess the incidence of uncommon adverse events (AEs) [2], which may be complicated further by inconsistent reporting practices [3,4]. Because infrequent AEs may be inadequately characterized or overlooked in early-phase studies, their relationship to treatment dose and/or schedule can remain undetermined.

Cholecystitis [5-10] and other gallbladder toxicities (including biliary colic, cholelithiasis, gallbladder enlargement, and gallbladder wall thickening/edema [7,8,11,12]) have been reported in clinical trials investigating motesanib, an orally administered small-molecule antagonist of vascular endothelial growth factor receptors (VEGFRs) 1, 2, and 3; platelet-derived growth factor (PDGFR); and Kit for the treatment of advanced solid tumors. Conversely, cholecystitis was not reported as an AE in other studies of motesanib as monotherapy [12,13] or combined with cytotoxic chemotherapy [14] or other agents [11,15,16]. However, it is unknown how many patients who received motesanib in these studies had undetected or underreported gallbladder toxicity, particularly given that abdominal pain was a frequently reported AE [5-8]. Thus, the proportion of patients with changes in gallbladder size and/or function is potentially greater than the incidence of gallbladder AEs. The etiology of gallbladder toxicity associated with motesanib treatment is uncertain, but it is interesting to note that cholecystitis has been reported among patients treated with other inhibitors of tyrosine kinases [17-26].

The previous clinical studies of motesanib suggested that a dosing regimen of 75 mg twice daily continuously may be associated with an increased risk of gallbladder toxicities. Therefore, to investigate more thoroughly the occurrence of gallbladder toxicity associated with motesanib treatment, we designed a randomized phase 1b study with three alternative motesanib dosing regimens to directly assess the effects of motesanib on both the size and function of the gallbladder using ultrasound and hepatobiliary iminodiacetic acid scan using cholecystokinin (CCK-HIDA), respectively.

## Methods

### Eligibility

Patients (≥18 years) had histologically confirmed advanced metastatic solid tumors; measurable or nonmeasurable disease per Response Evaluation Criteria in Solid Tumors (RECIST) [27] version 1.0; an Eastern Cooperative Oncology Group performance status ≤2; an in situ gallbladder at screening ultrasound; adequate cardiac, renal, hepatic, and hematologic function; and were ineligible to receive or had progressed on standard-of-care therapies. Key exclusion criteria were history of cholecystitis, prior biliary procedure, or prior or ongoing biliary disease; uncontrolled central nervous system metastases; uncontrolled hypertension (>150/90 mmHg); peripheral neuropathy grade >1; arterial/venous thrombosis within 1 year and bleeding diathesis or bleeding within 14 days and major or minor surgery within 28 days or 7 days, respectively, of randomization; radiation therapy within 14 days; active dosing with anticoagulation therapy (except prophylactic low-dose warfarin; heparin or heparin flushes); or prior treatment with small-molecule VEGFR inhibitors. Prior treatment with bevacizumab was permitted if the last dose was administered ≥42 days from randomization. Patients provided written informed consent. Study procedures were approved by an institutional review board at each site.

### Study design and treatment

In this open-label phase 1b study (11 sites in the United States and Australia), patients were randomized 2:1:1 to receive (in 21-day cycles) motesanib orally as follows: 125 mg once daily (QD; Arm A), 75 mg twice daily (BID) for 2 weeks followed by a 1-week treatment-free period (Arm B), or 75 mg BID for 5 days followed by a 2-day treatment-free period (Arm C). It was hypothesized that the treatment-free periods would prevent chronic inhibition of the VEGF axis, thus limiting adverse events that may otherwise be associated with continuous dosing. In each arm, up to eight additional patients (nonrandomly assigned) could be treated depending on the degree of variability in the primary endpoint measurements. Treatment continued until disease progression or unacceptable toxicity. Motesanib doses could be reduced (in 25-mg decrements) or withheld to manage toxicity; treatment could be resumed at the lower dose once toxicity had resolved (dose re-escalation was not permitted). Treatment was discontinued in patients requiring >2 dose reductions. Hypertension, thrombosis, gallbladder toxicity, and proteinuria were managed using protocol-specific guidelines.

The primary endpoints were mean change from baseline in gallbladder size (volume by ultrasound) and function (ejection fraction by CCK-HIDA). Secondary endpoints included mean change from baseline in gallbladder size (volume) by computed tomography (CT) scan, maximum change from baseline in gallbladder size (volume) and function (ejection fraction), changes in gallbladder dimensions other than volume (by ultrasound), assessment of gallbladder filling (by CCK-HIDA), change in gallbladder size and function between the last on-treatment and the last available off-treatment measurement, objective response, pharmacokinetics of motesanib, and incidence of treatment-emergent AEs.

### Assessment of gallbladder size and function

Gallbladder volume was assessed by ultrasound after a ≥8 hours fast at screening (within 21 days prior to randomization) and before the motesanib morning dose on days 8 and 15 of cycle 1, on day 1 of cycles 2 and 3, every 6 weeks thereafter, and at the safety follow-up (30 to 33 days after the last dose). Ultrasound was performed weekly when motesanib was withheld and weekly for 4 weeks following treatment discontinuation. Gallbladder ultrasound measurements were assessed by independent central radiologic review (MedQIA, Los Angeles, CA, USA). Gallbladder ejection fraction was assessed by investigators or other study site personnel using CCK-HIDA at screening (within 21 days of randomization), on day 1 of cycle 2, day 1 of cycle 6 (±3 days), and at the safety follow-up. Study-specific training and standard operating procedures were supplied to all radiology technicians.

### Tumor assessments

Tumor response per RECIST [27] was assessed by the investigators. Magnetic resonance imaging or CT scans were performed at screening, every 6 weeks thereafter, and at the safety follow-up. Complete or partial responses were confirmed >28 days after the initial response assessment throughout the study. Patients who discontinued without a postbaseline tumor assessment or confirmation were considered nonresponders.

### Adverse events

AEs occurring during treatment and through the safety follow-up were recorded and graded according to the National Cancer Institute Common Terminology Criteria for Adverse Events (version 3.0).

### Pharmacokinetic analysis

Blood samples were collected as follows: predose and at 30 minutes and 1, 2, 4, 6, 8, and 24 hours postdose on day 1 of weeks 1 and 4, and predose and 1 to 2 hours postdose at weeks 2, 3, and 7 and every 3 weeks thereafter. Noncompartmental analysis was performed on individual plasma motesanib concentrations from week 1 (day 1 of cycle 1) and week 4 (day 1 of cycle 2) using validated WinNonlin Enterprise software (Version 5.1.1, Pharsight Corporation, Mountain View, CA, USA) to estimate the maximum observed plasma concentration (C_max_), the observed minimum (trough) plasma concentration at 24 hours postdose (C_min_), and the area under the plasma concentration-time curve (AUC). Motesanib concentrations were assessed as described previously [14].

### Statistical analysis

The sample size was 48 patients. Assuming a standard deviation of 110cc and a one-sided 95% confidence interval (CI), a sample size of 24 patients for Arm A and 12 patients each for Arms B and C would allow for an estimate of the overall average change from baseline in gallbladder volume to within ±37cc and ±52cc, respectively. Patients were randomized 2:1:1.

The ultrasound and CCK-HIDA gallbladder analysis sets, which included all randomized patients who received ≥1 dose of motesanib and had baseline and ≥1 evaluable follow-up ultrasound or CCK-HIDA, respectively, were used for the principal analysis of endpoints related to gallbladder size and characteristics. For each dosing scheme, estimates for the mean and maximum change from baseline in gallbladder size (volume as measured by ultrasound) and function (ejection fraction as measured by CCK-HIDA scan) were calculated. Mean change from baseline was calculated by taking the difference between the baseline gallbladder measurement and the average gallbladder measurement observed during study treatment. The mean (95% CI) difference was then calculated across all patients for each treatment arm, and for the whole study population. Maximum change from baseline in gallbladder size or volume was calculated by taking the difference between the baseline gallbladder measurement and the maximum gallbladder measurement observed during study treatment. The mean (95% CI) maximum change from baseline was then calculated across all patients for each treatment arm, and for the whole study population. Reversibility of changes in gallbladder volume and ejection fraction were evaluated calculating changes between the last on-treatment measurement and the last available measurement following the discontinuation of motesanib. Covariates (treatment, age, sex, body mass index, and nonsteroidal anti-inflammatory drug [NSAID] use) were explored in a linear regression model for potential relationships with gallbladder volume. Objective response was assessed for the safety analysis set, including only patients with measureable disease at baseline.

## Results

### Patients

**Figure 1:**
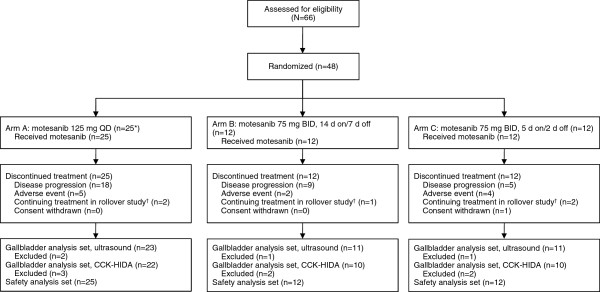
**Disposition of patients in the study.** *One patient was nonrandomly assigned to Arm A and received treatment with motesanib 125 mg QD. ^†^Total shown does not reflect 2 additional patients who discontinued motesanib for other reasons but later were granted a waiver to continue in a rollover study.

Between March 20, 2007, and December 12, 2008, 48 patients were randomized to treatment with motesanib at three different doses: Arm A (125 mg QD), n = 24; Arm B (75 mg BID 2 weeks on/1 week off), n = 12; Arm C (75 mg BID 5 days on/2 days off), n = 12 (Figure 1). As permitted per protocol, one additional patient was nonrandomly assigned to Arm A for a total enrollment of 49 patients; all received ≥1 dose of motesanib. Thyroid cancer was the most common tumor type (Table 1). Demographics and baseline characteristics were generally balanced among the treatment arms, although fewer patients received prior therapies in Arm A than in Arms B and C (Table 1). The ultrasound gallbladder analysis set included 92% of patients; the CCK-HIDA gallbladder analysis set included 84% of patients. One patient (Arm A) with mesothelioma had a cholecystectomy during the study (see *Adverse Events*) but had baseline and evaluable postbaseline assessments and was therefore included in both gallbladder analysis sets. All patients discontinued treatment (Figure 1). Twenty patients (80%) in Arm A, 8 (67%) in Arm B, and 8 (67%) in Arm C completed the safety follow-up. Reasons for not completing the safety follow-up were disease progression (Arms A and C, n = 1 each), death (Arm A, n = 2; both due to disease progression), AE (Arm C, n = 1), and withdrawn consent (Arm B, n = 1). Median follow-up times in Arms A, B, and C were 17 (range, 6–57), 18 (1–58), and 22 (5–60) weeks, respectively.

**Table 1:** Patient demographics and baseline characteristics

**Characteristics**	**Arm A**	**Arm B**	**Arm C**	**All patients**
	**Motesanib**	**Motesanib**	**Motesanib**	
	**125 mg QD**	**75 mg BID 2 wk on/1 wk off**	**75 mg BID 5 d on/2 d off**	
	**n = 25**	**n = 12**	**n = 12**	**N = 49**
Sex, n (%)				
Women	10 (40)	6 (50)	5 (42)	21 (43)
Men	15 (60)	6 (50)	7 (58)	28 (57)
Race, n (%)				
White	22 (88)	11 (92)	11 (92)	44 (90)
Black	2 (8)	0 (0)	0 (0)	2 (4)
Hispanic	1 (4)	1 (8)	0 (0)	2 (4)
Native Hawaiian or other Pacific Islander	0 (0)	0 (0)	1 (8)	1 (2)
Median age (range), y	59 (28–70)	52 (30–70)	59 (22–81)	58 (22–81)
Age group, n (%)				
<65 y	18 (72)	10 (83)	9 (75)	37 (76)
≥65 y	7 (28)	2 (17)	3 (25)	12 (24)
≥75 y	0 (0)	0 (0)	1 (8)	1 (2)
Tumor type, n (%)				
Thyroid	1 (4)	7 (58)	4 (33)	12 (24)
Colon	3 (12)	0 (0)	1 (8)	4 (8)
Non–small-cell lung	3 (12)	0 (0)	1 (8)	4 (8)
Carcinoma of unknown origin	1 (4)	1 (8)	0 (0)	2 (4)
Cervix	1 (4)	0 (0)	1 (8)	2 (4)
Oral	1 (4)	0 (0)	1 (8)	2 (4)
Ovarian	1 (4)	0 (0)	1 (8)	2 (4)
Small-cell lung	0 (0)	2 (17)	0 (0)	2 (4)
Soft tissue sarcoma	1 (4)	0 (0)	1 (8)	2 (4)
Bile duct	1 (4)	0 (0)	0 (0)	1 (2)
Bone sarcoma	1 (4)	0 (0)	0 (0)	1 (2)
Esophageal	1 (4)	0 (0)	0 (0)	1 (2)
Kidney	1 (4)	0 (0)	0 (0)	1 (2)
Liver	1 (4)	0 (0)	0 (0)	1 (2)
Squamous cell carcinoma of head and neck	1 (4)	0 (0)	0 (0)	1 (2)
Other	7 (28)	2 (17)	2 (17)	11 (22)
ECOG performance status, n (%)				
0	14 (56)	8 (67)	9 (75)	31 (63)
1	10 (40)	4 (33)	3 (25)	17 (35)
2	1 (4)	0 (0)	0 (0)	1 (2)
Disease stage, n (%)				
Stage III	1 (4)	0 (0)	0 (0)	1 (2)
Stage IV	22 (88)	11 (92)	11 (92)	44 (90)
Unknown	2 (8)	1 (8)	1 (8)	4 (8)
Number of sites of disease,* n (%)				
0	1 (4)	0 (0)	1 (8)	2 (4)
1	13 (52)	4 (33)	3 (25)	20 (41)
2	10 (40)	5 (42)	6 (50)	21 (43)
≥3	1 (4)	3 (25)	2 (17)	6 (50)
Number of prior therapies,^†^ n (%)				
0	5 (20)	1 (8)	1 (8)	7 (14)
1	5 (20)	1 (8)	2 (17)	8 (16)
2	2 (8)	1 (8)	3 (25)	6 (12)
≥3	13 (52)	9 (75)	6 (50)	28 (57)
Alcohol use, n (%)				
Never	1 (4)	5 (42)	4 (33)	10 (20)
Former	5 (20)	1 (8)	2 (17)	8 (16)
Current	18 (72)	5 (42)	5 (42)	28 (57)
Missing	1 (4)	1 (8)	1 (8)	3 (6)

BID = twice daily; ECOG = Eastern Cooperative Oncology Group; QD = once daily.

*Sites of disease as assessed by investigator.

^†^Prior therapies include all cancer therapies before study enrollment.

### Effects of motesanib dose on gallbladder size and function

**Figure 2:**
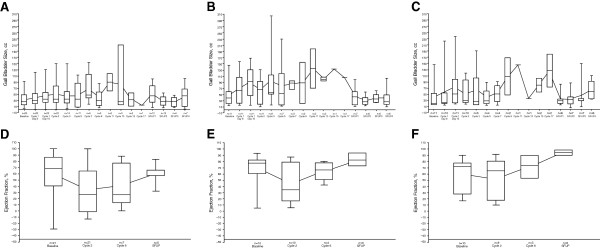
**Change in gallbladder size and function.** Mean (dots connected by lines) and median (25^th^ and 75^th^ quartiles; solid horizontal lines) gallbladder size (**A**, **B**, **C**) and function (**D**, **E**, **F**) over time per independent review in Arms **A**, **B**, and **C**, respectively. Error bars represent the minimum and maximum values. SFUP, safety follow-up.

Baseline gallbladder volume and ejection fraction were similar across arms (Table 2). Across all patients, gallbladder volume increased by a mean 22.2 cc (median, 17.3 cc; range, −43.3 to 83.2 cc) from 38.6 cc at baseline during motesanib treatment. Gallbladder volume increased from baseline in all dosing cohorts, starting before the end of the first 21-day motesanib treatment cycle (Table 2; Figure 2A, B, C).

**Table 2:** Gallbladder Volume (per Independent Review) and Ejection Fraction (per Investigator)

**Endpoint**	**Arm A**	**Arm B**	**Arm C**
	**Motesanib**	**Motesanib**	**Motesanib**
	**125 mg QD**	**75 mg BID 2 wk on/1 wk off**	**75 mg BID 5 d on/2 d off**
	**n = 23**	**n = 11**	**n = 11**
Gallbladder volume, cc (95% CI)	n = 23	n = 11	n = 11
Baseline	33.3 (22.5–44.1)	48.1 (23.1–73.1)	40.2 (14.1–66.2)
Mean change from baseline	17.7 (6.4–28.9)	26.8 (11.5–42.1)	26.9 (8.8–45.1)
Maximum change from baseline	45.6 (20.2–70.9)	74.4 (41.3–107.4)	67.3 (30.8–103.8)
Gallbladder ejection fraction, % (95% CI)	n = 21	n = 10	n = 10
Baseline	59.1 (43.5–74.8)	68.7 (50.5–87.0)	58.5 (38.4–78.6)
Mean change from baseline	−24.1 (−38.2 to −9.9)	−25.0 (−43.9 to −6.1)	−3.3 (−25.0 to 18.4)
Maximum change from baseline	−30.1 (−46.4 to −13.7)	−26.5 (−45.0 to −8.0)	−6.5 (−29.8 to 16.8)
Reversibility of gallbladder volume changes, cc (95% CI)	n = 16	n = 9	n = 8
Mean change in gallbladder volume after discontinuation of motesanib	−8.5 (−38.8 to 21.7)	−16.2 (−37.4 to 5.1)	−7.4 (−67.1 to 52.4)
Mean change in gallbladder volume from baseline to last available off- treatment measurement	10.4 (−10.0 to 30.8)	−14.4 (−31.1 to 2.4)	7.1 (−28.9 to 43.0)
Reversibility of gallbladder ejection fraction changes, % (95% CI)	n = 5	n = 3	n = 2
Mean change in ejection fraction after discontinuation of motesanib	10.8 (−45.8 to 67.4)	63.0 (24.0 to 102.0)	46.0 (−347.9 to 439.9)
Mean change in ejection fraction from baseline to last available off- treatment measurement	−16.6 (−53.3 to 20.1)	7.7 (−3.8 to 19.1)	14.5 (−55.4 to 84.4)

BID = twice daily; QD = once daily.

Motesanib treatment also affected gallbladder function. Across all patients, ejection fraction decreased by a mean 19.2% (median, −18.0%; range, −81% to 67%) from 61.3% at baseline during the study. Gallbladder ejection fraction during treatment was generally lower than baseline measurements (Table 2; Figure 2D, E, F).

Changes in gallbladder volume and function appeared to be at least partially reversible. Among 45 patients in the gallbladder volume analysis set, 33 had an evaluable ultrasound after motesanib discontinuation. In each arm, mean changes from last on-treatment to last available off-treatment measurement indicated a decrease in gallbladder volume (Table 2). Similarly, among the 41 patients in the gallbladder ejection fraction analysis set who had an evaluable CCK-HIDA after motesanib discontinuation (n = 10), gallbladder mean ejection fraction increased between these two time points (Table 2).

To adjust for potential confounding factors, linear regression analyses were performed. The results were consistent with the data from the preplanned analysis, showing a trend toward decreasing gallbladder volume and increasing gallbladder ejection fraction over time (data not shown).

Treatment, age, sex, body mass index, and NSAID use were examined in a linear regression model as potential covariates for gallbladder volume. Of those, only NSAID use was positively associated with increased gallbladder volume as assessed by ultrasound (*P* = .0133); the other covariates were not significantly associated with gallbladder volume. Exploratory analyses did not show an association between pharmacokinetic exposure to motesanib and gallbladder volume (data not shown). Covariate analyses and exploratory pharmacokinetic exposure analyses for gallbladder ejection fraction could not be performed because of insufficient ejection fraction data.

### Changes in other gallbladder characteristics

Some patients in Arms A and B developed gallstones and/or pericholecystic fluid while receiving motesanib (Table 3), including two patients who developed *de novo* gallstones; however, two patients with gallstones at baseline did not have gallstones at subsequent examinations. Sludge occurred in all three treatment arms at relatively high incidence rates (Arms A/B/C, 39%/36%/27%).

**Table 3:** Specific gallbladder findings (per Independent Ultrasound Review)

	**Arm A**			**Arm B**			**Arm C**		
	**Motesanib 125 mg QD**			**Motesanib 75 mg BID**			**Motesanib 75 mg BID**		
				**2 wk on/1 wk off**			**5 d on/2 d off**		
**Patient incidence, n (%)**	**Baseline**	Post baseline*	**Post treatment**^**†**^	**Baseline**	Post baseline*	**Post treatment**^**†**^	**Baseline**	Post baseline*	**Post treatment**^**†**^
	**(n = 23)**	**(n = 23)**	**(n = 16)**	**(n = 11)**	**(n = 11)**	**(n = 9)**	**(n = 11)**	**(n = 11)**	**(n = 8)**
Gallstones	3 (13)	4 (17)	3 (19)	3 (27)	2 (18)	2 (22)	0 (0)	0 (0)	0 (0)
Sludge	0 (0)	9 (39)	4 (25)	0 (0)	4 (36)	4 (44)	0 (0)	3 (27)	0 (0)
Pericholecystic fluid	0 (0)	1 (4)	0 (0)	1 (9)	1 (9)	0 (0)	0 (0)	0 (0)	0 (0)
Common duct dilation	0 (0)	0 (0)	0 (0)	0 (0)	0 (0)	0 (0)	0 (0)	0 (0)	0 (0)

*Data within the table indicate number of patients with at least one incidence of the specific gallbladder findings listed at any point during postbaseline treatment.

^†^Data within the table indicate number of patients who had the specific gallbladder findings listed at their last available off-treatment assessment.

### Adverse events

Adverse events considered related to treatment with motesanib by investigators were generally consistent in frequency and severity with what has been reported in previous motesanib studies [5,7-9,12,14,15]. Incidence of grade ≥3 treatment-related AEs in Arms A, B, and C was 32%, 42%, and 33%, respectively. Two patients had grade 4 AEs (one each in Arms B and C). Two deaths occurred during the study; both were caused by disease progression.

Gallbladder toxicity events (all considered treatment-related) occurred only in Arm A (n = 6, 12%). Three patients had cholecystitis that resolved after motesanib treatment was permanently discontinued. One event was of grade 1 and resolved within 1 week while motesanib was withheld. One event was of grade 2 and occurred approximately 1 month after the last motesanib dose; it resolved 2 months later. A 70-year-old white man with metastatic non–small-cell lung cancer developed grade 3 cholecystitis that was managed without surgery. Symptoms appeared approximately 3 weeks after initiation of motesanib, with ultrasound showing gallbladder distension and the presence of sludge. CCK-HIDA revealed a patent cystic duct and gallbladder dyskinesia. The patient discontinued motesanib and was treated with oxycodone and paracetamol. Three weeks later, CCK-HIDA measurements were normal and the symptoms had resolved. One patient, a 56-year-old white man with stage IV mesothelioma, had serious grade 3 acute cholecystitis resulting in cholecystectomy. The event occurred approximately 1 month after treatment initiation. At the time of hospitalization, the patient had a 24-hour history of right upper quadrant pain; Murphy’s sign was positive on abdominal examination. Motesanib was withheld, and ultrasound revealed gallbladder distension, wall thickening (4.4 cm), intramural edema, mural hypervascularity, trace of pericholecystic fluid, and no biliary tract dilation. Cholecystectomy was performed 8 days after cessation of motesanib, and the patient resumed motesanib treatment 11 days later. At the safety follow-up, two patients had ongoing grade 1 gallbladder disorders, specifically gallbladder dysfunction and gallbladder wall thickening, with the latter prompting a dose reduction. Twelve patients had right upper quadrant pain during the study (Arms A/B/C, n = 8/1/3); these events occurred at variable times after initiation of motesanib. However, the available data do not help distinguish between pain due to gallbladder toxicity versus other etiologies, such as liver metastases.

### Objective response

Most patients had measureable disease at baseline (Arm A, n = 24 [96%]; Arm B, n = 12 [100%]; Arm C, n = 11 [92%]). No complete responses were achieved, but one patient with stage IV thyroid cancer in Arm B had a confirmed partial response (overall objective response rate, 2%). Twenty-eight patients (60%) had stable disease as best tumor response (Arm A, n = 15 [63%]; Arm B, n = 6 [50%]; Arm C, n = 7 [64%]), with durable (≥24 weeks) stable disease in 8 (17%) patients (Arm A, n = 6 [25%]; Arm B, n = 1 [8%]; Arm C, n = 1 [9%]). Fifteen patients (32%) had progressive disease (Arm A, n = 8 [33%]; Arm B, n = 3 [25%]; Arm C, n = 4 [36%]).

### Pharmacokinetics

Motesanib was rapidly absorbed, and there was no evidence of drug accumulation after QD administration. The median C_max_ values in Arms A, B, and C were 630, 323, and 355 ng/mL, respectively; the median C_min_ values were 14, 60, and 35 ng/mL, respectively. In Arm B, the median motesanib concentration after the 1-week wash-out period was <0.2 ng/mL (the limit of quantitation); in Arm C, the median motesanib concentration after the 2-day wash-out period was 1.2 ng/mL. The median AUC values estimated from the three dosing regimens appeared similar, ranging from 1.9 to 3.0 μg·hr/mL.

An exploratory analysis investigated the potential relationship between drug exposure (C_max_, C_min_, and AUC) and change in gallbladder size. The results showed no consistent trend between gallbladder size and motesanib exposure.

## Discussion

In this randomized phase 1b study designed to assess gallbladder-related toxicity among patients receiving three motesanib dose schedules, increased gallbladder volume, decreased gallbladder function, and other gallbladder changes, including development of gallstones and sludge, were common. Changes in gallbladder volume were observed as early as in the first cycle of motesanib treatment. Symptomatic gallbladder toxicity occurred in six patients, one of whom had acute cholecystitis requiring a cholecystectomy. Other toxicities were generally consistent with those reported in previous motesanib studies and for the class of VEGF pathway inhibitors. While increases in gallbladder volume and decreases in gallbladder function did not appear to be dose- or schedule-dependent, gallbladder toxicity occurred only in Arm A (motesanib 125 mg QD).

Gallbladder toxicity, at varying incidence rates, has been described in most motesanib studies [5,7,8,10,28]; however, considering the findings summarized herein, gallbladder-associated AEs may have been underdetected. This may particularly apply to earlier-conducted studies that reported no [12-16] or low [5,9,28] incidence rates of cholecystitis (but no other gallbladder toxicity) and to patients who presented only with right upper quadrant pain along with other possible reasons for pain, including liver metastases. For example, Sawaki and colleagues described the incidental discovery by ultrasound of extended gallbladder or wall thickening in three patients [12]. Given that many VEGF pathway inhibitors block the same or similar targets as motesanib (Table 4), and because of the incidence of abdominal pain with tyrosine kinase inhibitors [17-26,29-37], changes in gallbladder size and function not manifested as symptomatic toxicity may occur more frequently during treatment with these agents than generally believed. The results of our study should encourage investigators to more closely examine potentially gallbladder-related symptoms in studies of VEGF pathway inhibitors and among patients treated outside of clinical trials.

**Table 4:** Gallbladder-related toxicity and potential gallbladder-related toxicity reported with tyrosine kinase inhibitors other than motesanib

**Agent**	**Molecular target(s)**	**Study / Study type**	**Adverse events reported**
Cediranib	VEGFR1, VEGFR2, VEGFR3	Laurie et al. [21] – phase 1 study	Acute cholecystitis
		Batchelor et al. [22] – phase 2 study	Gallbladder obstruction, abdominal pain
Imatinib	BCR-ABL, Kit, PDGFR-α, PDGFR-β	Yeh et al. [23] – single-arm study	Gallstones
		Breccia et al. [37] – case report	Gallstones, gallbladder wall thickening, abdominal pain
		Grant et al. [24] – phase 1 study	Cholecystitis
Sorafenib	VEGFR1, VEGFR2, VEGFR3, Raf, PDGFR-β, Flt-3, Kit	Sanda et al. [20] – case report	Right upper abdominal pain, gallbladder edema, acute acalculous cholecystitis
		Nexavar European public assessment report [26]	Cholecystitis, cholangitis
		Nexavar US prescribing information [25]	Cholecystitis, cholangitis
Sunitinib	VEGFR1, VEGFR2, VEGFR3, PDGFR-α, PDGFR-β, Flt-3, Kit	Motzer et al. [17] – single-arm study	Acute cholecystitis
		De Lima Lopes, Jr., et al. [18] – case report	Acute emphysematous cholecystitis, right upper abdominal pain, gallbladder distension
		Gomez-Abuin et al. [19] – case report	Acute acalculous cholecystitis, right upper abdominal pain, gallbladder wall thickening

ALT = alkaline phosphatase; PDGFR = platelet-derived growth factor receptor; VEGFR = vascular endothelial growth factor receptor.

The biologic mechanisms that underlie the gallbladder changes associated with motesanib treatment are not yet elucidated. The toxicity may be related to antiangiogenic activity of motesanib in the gallbladder which could be exacerbated by accumulated motesanib, considering the drug’s biliary excretion pattern (Amgen Inc., data on file). Accumulation of motesanib within the gallbladder following the excretion (and reactivation) of its major metabolite, motesanib glucuronic acid [38], in the relatively high pH of the bile may result in irritation to the gallbladder or possibly even transient ischemia with subsequent sludge accumulation, transient obstruction, pain, and ultimately, cholecystitis or cholecystitis-like symptoms. One potential solution may be to avoid conditions that are known to reduce gallbladder emptying such as fasting and low-fat diets. Consideration should also be given to the possibility that gallbladder toxicity is an on-target effect of inhibition of one or more of the molecular targets of tyrosine kinase inhibitors.

The design of this study may be appropriate for investigating gallbladder toxicity with other investigational agents, including tyrosine kinase inhibitors. The measured changes from baseline in gallbladder volume and ejection appeared to be both robust and greater than anticipated inter- or intrapatient variance. In Arms A and B, the 95% Cl for the mean and maximum changes from baseline did not encompass zero, and the observed changes were consistent with differences between patients with gallbladder disease and healthy control participants reported in previous studies [39,40]. Thus, the results demonstrate that, when coupled with rigorous quality control/assurance procedures and training, routine diagnostic techniques (eg, ultrasound, CT, and CCK-HIDA [41]) can be used to evaluate the incidence and timing of gallbladder toxicity assessed as changes in volume, ejection fraction, and filling, and to identify other abnormalities, such as gallstones and pericholecystic fluid. Better characterization of these risks is important because of the potential seriousness of gallbladder toxicity. More broadly, targeted assessments of specific AEs may help characterize the toxicity of investigational cancer therapeutics. The study was limited by the lack of a placebo arm, and the small sample size potentially restricted AE and other assessments.

## Conclusions

In conclusion, motesanib monotherapy was associated with increased gallbladder volume and decreased ejection fraction in most patients, regardless of dosing regimen and exposure, which appeared to be at least partially reversible. Motesanib had a toxicity profile consistent with previous studies. The etiology of gallbladder toxicity during motesanib treatment remains uncertain.

## Competing interests

LSR, LL, NDB, and JJS have no competing interests to declare. TJP and LJW have been consultants to Amgen Inc. RVB has received honoraria from and holds stock in Amgen Inc. HIH has received research funding from GSK. NCT has received research funding from Amgen Inc. and has provided expert testimony on behalf of Amgen Inc. SM, Y-JH, and C-PH are employees of and shareholders in Amgen Inc.

## Authors’ contributions

LSR, HIH, and Y-JH participated in conception and design of the study. LL, LSR, TJP, NDB, HIH, JJS, LJW, SM, C-PH, and NCT participated in collection and assembly of data. LSR, RVB, HIH, LJW, SM, Y-JH, and C-PH participated in data analysis and interpretation. All authors participated in writing or revising the manuscript and provided their approval of the final version of the manuscript.

## Pre-publication history

The pre-publication history for this paper can be accessed here:

http://www.biomedcentral.com/1471-2407/13/242/prepub
